# Gout in Hospitals

**Published:** 1894-05-05

**Authors:** John Beddoe

**Affiliations:** late Physician to the Royal Infirmary, Bristol


### GOUT IN HOSPITALS.

By John Beddoe, M.D., LL.D., F.R.S., late Physician to the Royal Infirmary, Bristol.

It used to be said that gout was a very rare disease in hospitals, but such is no longer the case nowadays. Nor is it peculiar to the upper classes, nor unknown in Scotland; in fact the general statements made about it are almost all liable to more or less exception. I have known as many as four cases of gout to be lying at one time, not only in one hospital, but in one ward thereof.

Here is a typical and instructive case: T. D., mason, aged 52, a fine robust ruddy man, intelligent, and pleasant looking. "How have you been accustomed to live, D.?" "Oh! very well and very regular, sir! My missus always takes good care of me." "You are married then? Any children?" "No, sir! never had none." "What do you drink?" "Beer, sir; but not very much." "How much, and how often?" "Well, sir; half a pint or a pint at eleven o'clock, and a pint at dinner, and another pint at supper; nothing more."

Here was a respectable decent man, a good workman, and therefore always in full employment and earning good wages, which were spent in good feeding, plenty of meat, and just enough beer to help in the production of uric acid and waste products, but not enough to disorder his health in other ways or to diminish the keen appetite engendered by moderate labour in the open air, and stimulated by the cookery of a wife who had no children to divert her attention or her money from her husband. A little colchicum and alkali soon set this man on his feet; but more valuable, probably, was the advice he got to eat in the future less meat, and more milk and vegetables, and to limit his beer strictly to half a pint.

Presently we come to another case, in some respects almost identical with the former. This man exercises the healthiest of handicrafts; he is a gardener, aged 58, with the same good physique and sanguine complexion as the other man. He also has lived comfortably, and confesses to from two to three pints of beer daily; perhaps the actual quantity may have been a little more. He has had a yearly attack ever since his first one, which was 27 years ago. Sometimes they came in the winter, sometimes in the summer, and he has noticed that the summer attacks are the more severe and protracted. On the ordinary chemical theory, this is easily explicable; when the attack is postponed till summer the interval is longer and the accumulation of gouty poison is greater.

Long before Sir Alfred Garrod developed his theory of gout, a theory so beautifully simple that for years no one dared to raise an objection to it, experience had taught those who were most concerned, that the conjunction of excess in animal food with indulgence in liquors, especially beer and sweet wines, was most surely conducive to gout. And we shall do well if we remember this cardinal doctrine, notwithstanding the heretics among us who not unfrequently prescribe port wine to the gouty. On the whole red wines are more apt to excite paroxysms of the disease than white; thus Bordeaux occasionally and Burgundy very frequently disagrees, whereas Liebig boldly affirmed that along the Rhine, where wine indeed is drunk in plenty, but almost exclusively white wine, gout was in his time practically unknown. This statement was, I dare say, too broadly positive; but certainly Rhenish wines in moderate quantity may generally be prescribed to the gouty with some confidence. Sir William Roberts seems to think that red wines exercise a slight retarding action on primary digestion, and that their superior wholesomeness as regards the nervous system is in some degree due to this fact, as the contained alcohol is more slowly absorbed, and produces a less sudden and marked stimulation and reaction. Be that as it may, it is pretty clear that the best regimen for the gouty is one that will promote the easy and perfect digestion of food, and the rapid and complete evacuation of waste products. Thus, "αριστον μεν ὑδωρ"; but in the pretty numerous cases in which digestion does not go on well without the aid of a little stimulant, it is better to make the concession. The slight diuretic action which whiskey appears to exert, may have something to do with the beneficial influence with which it is credited.

But why should sugar, or at all events drinks qualified with sugar, prove specially pernicious? Sugar does not contain the materials necessary for the production of uric acid; no more, indeed, does alcohol. If, therefore, it be true that either of them alone, and yet more decidedly the two in conjunction, do favour the production or deposition of uric acid, it must be by their action, or that of results of their metamorphosis, on some nitrogenous substance or substances. Again, why should sugar be so much blamed, while starch, which must perforce become sugar in the animal economy, escapes without censure? I may perhaps be permitted to make one or two suggestions, though I have not at present space to follow them up.

It may be that both alcohol and sugar act as gout producers by virtue (or vice) of their antitriptic character. Easily oxidisable, they may prevent or delay the action of oxygen on nitrogenous substances which are in course of breaking down; they may seduce, as it were, the oxygen from its duty, and may leave time and opportunity for the formation of pernicious products. And though starch really has to pass through a stage in which it exists as glucose, yet the metamorphosis is slow and gradual, and at no one time may the portal vessels be so much engorged as may be the case after a meal largely saccharine, and therefore rapidly absorbed.

The popular idea, however, is that sugar is injurious because, or when, it "turns acid" in the early stages of digestion—under the same conditions, in short, as those in which heartburn is produced, And this, dyspeptics tell us, is particularly apt to occur after the ingestion of a quantity of dilute saccharine fluid, but much less so where sugar has been a principal constituent of a solid meal.

